# Azoturia

**Published:** 1897-07

**Authors:** John R. Hart

**Affiliations:** Philadelphia


### AZOTURIA.

By John R. Hart, V.M.D.,
PHILADELPHIA.

(Concluded from page 386.)

*Treatment.* Treatment presents to the veterinarian two grand problems for his solution; first, to discover its nature, and, second, to devise its cure. A knowledge, therefore, of the disease examined, as well as those with which it may be confounded, is essential.

Diagnosis lies before us as the basic principle of the practice of medicine. An intelligent conception of a disease is a prerequisite to the application of the medicines necessary to effect its removal, and also to prevent confounding it with others whose resemblance to the particular disease makes it difficult to distinguish them. Its complications must be guarded against; its character, which subjects it to various accidental influences, must be carefully considered and thoroughly known. The man who is equipped with such knowledge, with the principles applicable, and with sound discretion and judgment enjoys immeasurable advantages over him who lacks them, or whose mind is so preoccupied by theory as to make intelligent treatment a difficult if not impossible task. The treatment in one stage may be of no avail in the other. This is plainly demonstrated by the consensus of opinion of the different veterinarians in their modes of treatment. Some bleed with success, while others condemn this practice; some purge in all cases, some in only a few. With a success that varies, some treatment may be in place and some most decidedly out of place. The per cent. of recoveries should demonstrate and guide as to the proper mode of treatment.

There was a time when veterinarians carried a ball of aloes and calomel with them when called to a case that showed a disposition to constipation, with sluggish gait or stiffness in the limbs, as the owner put it. The ball was given with most excellent results and often no other treatment was required. This was the time when one or two balls were given once or twice yearly. While it was an old rut, in my mind it was a good one that would perhaps be well to practise in this day of theory.

While some animals will recover under any and all circumstances in a great many diseases, as they seem to have immunity from death, as they have immunity at times from the effects of the wrong treatment, as here is a treatment in azoturia and case reported as making a good recovery.

℞.—Aloes . . . . . . . . . ℥vij.
Hyd. Chlo. Mit. . . . . . . . ℨj.

With tincture of opium, to be given as often as necessary; to relax the bowels, is the duty of one, while the other is to constipate. While it is known that tincture of opium will increase the secretions of the skin, it will dry up all other secretions in the body and will produce, if continued repeatedly, cerebral convulsions.

In regard to purgative medicines in this disease, I assure you that I have had a larger per cent. of recovery among animals that did not respond to the action of a cathartic than I had from those which responded promptly to the cathartic. It was my custom to give to all the animals. This led me to consider well and finally to discontinue their use, with much better results, as the fecal matter had done all the harm it could possibly do at the time the animal was taken with the disease; our aim should be to get rid of the various poisons that the system is overloaded with.

Bleeding, as pursued in the human family, is resorted to when there may be an overloaded capillary circulation in diseases of the brain and spine, or at the point of uræmic convulsions, with great success; in other stages of disease similar to this bleeding would more quickly kill, perhaps, or if there was a tendency to clot in the circulation bleeding would be hurtful and dangerous.

Now as to treatment. The one point most to be observed in the treatment not only of azoturia, but in all diseases, is to know the pathological condition of the animal when seen. As we all know that all medicines are classified, it is not supposed that of these medicines in that particular class that we desire to use in the disease that they are classed to remedy. It is more important to know the stage the animal is in, as what would have the desired effect in the acute or inflammatory condition would not only be useless in the second stage, but would aggravate the disease, perhaps, like bleeding.

While a cathartic in one stage would be the proper remedy, in the next stage it might induce irritation that would kill the animal. The oil laxatives would be more beneficial. If they do no good, they would do no harm, but would have a nutritive effect. The question may be asked, Why give oil for nutritive effect, when it was too much feeding that caused the disease? The answer is that the very good living that assisted in causing the disease and in weakening the alimentary canal that nourishment should be administered which would be quickly absorbed. Experiments show that large amounts of oils given to animals leave no trace after death, while a cathartic too often aggravates the irritation already existing and would remove water from the blood—they would dehydrate the blood and consequently the tissues. In some cases cathartics will answer, but I would consider aloes out of place in any congested condition of the bowels, as it would increase the irritation. The most useful remedy for a congested liver or kidney is calomel, as it stimulates the ends of the secretory nerves in the cells, as well as relieves the overloaded capillaries, and will diminish putrefaction and eliminate urea. It may be used in any inflammation, as it is eliminated by all secretions, or calomel with soda bicarb., and continue the soda bicarb. in tablespoonful doses every two or three hours in the drinking-water. We, of course, know that the blood is alkaline, and that one of the poisons that is circulating in the blood in this disease is acid in excess. Any alteration from its natural constituent would cause a chemical change in the blood. Now, to overcome this condition, what is the next best thing to do? if again to alter by a chemical change, we would here use soda bicarb., as the soda bicarb. will eliminate uric acid from the blood. I would recommend small doses of the soda bicarb. in conjunction with the other treatment of diuretics, as too much medicine of an alkaline action may cause other chemical reactions, while the smaller doses would neutralize the acid in excess as well as assist digestion and absorption. The alkalies insure the gradual oxidation of the organic constituents of the fluids. They are also eliminated by the kidney and are antacids.

The diuretics most needed are those that act more on the kidney through the circulation, as by so doing you remove the uric acid and effete material that is circulating in the blood. Of this class I would recommend buchu, juniper spt. æth. nit., are the sedative class, as they stimulate the ends of the vaso-constrictor nerves in the vessel walls. If you have uræmia, the salts of potassa should not be used as a diuretic, as these salts determine intoxication, while the sodium would be used with safety. Where there is much excitement I have used in conjunction with diuretics fld. ext. hyoscyam. It is soon eliminated by the urine, it will also destroy the irritation that arises in the bladder. In these cases hyoscyamus acts as a hypnotic and will assist in destroying fibrin and acts very quickly on the nervous system as a sedative. Chloral hydrate is good in over-excitability or with a tendency to convulsions. Do not repeat too often. Chloroform inhaled will have the same effect in controlling the convulsion. Fld. ext. colch. I have used with success in small doses, after the bowels respond, as colch. in the over-fed or torpor of portal circulation, or where we have constipation and deficient excretion of the liver. Colch. will relieve by setting up an elimination process. It will also eliminate uric acid. Strychnine should not be used in paralysis of this kind. Its use is injurious in acute cases involving any alteration of the spinal cord, as it softens the spinal cord and causes the animal to die in convulsious. If you use it in early rigidity, it is without avail; if too late, there is fatty degeneration of the muscles. It is good only when the paralyzed limbs are completely relaxed.

Ergot is good in late stages where there is a tendency to congestion of the spinal cord or the meninges.

I use mustard over the loins in all cases. I have never found slings of any use, as they seem to excite the animal. As it does not seem common sense to hang an animal in this disease to cure him, I do not use them. With a good bed under him I allow the animal to lie down and turn him from side to side every two or three hours. When the urine does not clear up in two or three days it is not favorable. If the urine does clear and is stringy, there is a possibility of albumin or some complication in the spinal cord.

If we would instruct our clients in a system of feeding their animals and how to care for them while at rest, we would save ourselves considerable annoyance and trouble as well as gain a reputation and the full confidence of our clients.

They should be instructed in all matters pertaining to the stable, as I do not think there is one in our profession who is guilty of congratulating himself when called to attend an animal on the street suffering with azoturia. I have charge of a few large stables and cases of azoturia in them are rare. The animals are given frequently in their feed Glauber salt with a light diet twice weekly. All these animals are in good condition. I think Glauber salt will prevent effete matter from collecting in the blood.

I think if some of our colleges would take an animal or two and allow them to stand for one or two weeks as may be required, and feed to them the full complement of food at each meal; then weigh the excretion, test the urine at intervals, then change the food as circumstances may require to find out what composition in food that is retained brings on this poisonous condition, compare the income with the outgo, the ingesta with the excreta, they would learn not only what part of the ingesta is retained in the body, but would detect substances in the excreta not present in the food, and would learn all the changes which the body undergoes under the influence of the food.

It is possible, and would it not be wise to first discover what the muscles have lost of their nutritive elements, then increase this loss to the muscles by treatment identical with what is lost to give them tone?

At our next annual meeting, if not prevented by some unforseen cause, I will endeavor to give a detailed account of the conditions of organs and what is found, as well as conditions of blood and what is found with such assistance as may be rendered by the division of bacteriology.

